# Bladder Diverticulum—A Case Report

**DOI:** 10.21980/J8635C

**Published:** 2020-10-15

**Authors:** Savannah Tan, Sangeeta Sakaria

**Affiliations:** * University of California, Irvine, Department of Emergency Medicine, Orange, CA

## Abstract

A bladder diverticulum can be the consequence of a congenital abnormality or acquired as a result of trauma, infection, or outlet obstruction. Many are asymptomatic, but some may present with complications such as urinary tract infection, hematuria, or urinary retention.

A 76-year-old male presented to the emergency department (ED) for the second visit in one week with a chief complaint of urinary retention and lower abdominal pain. He had not voided since the prior night, when he had presented to the ED for the same compliant. During his initial visit, his symptoms were relieved by insertion of an in-&-out foley catheter. Point of Care Ultrasound (POCUS) of the bladder showed the appearance of two enlarged vertically aligned “bladders” with a central connection, concerning for a bladder diverticulum. Patient’s cause of bladder diverticulum was found to be secondary to outlet obstruction, specifically benign prostatic hypertrophy (BPH). An indwelling foley catheter was inserted, and the patient was discharged home with instructions for urology follow up.

The purpose of this report is to describe an anatomical anomaly of a bladder diverticulum presenting incidentally on Point of Care Ultrasound during routine workup of urinary retention.

**Topics:**

Urinary bladder diverticulum, urinary retention, benign prostatic hypertrophy, POCUS, case report.

**Figure:**
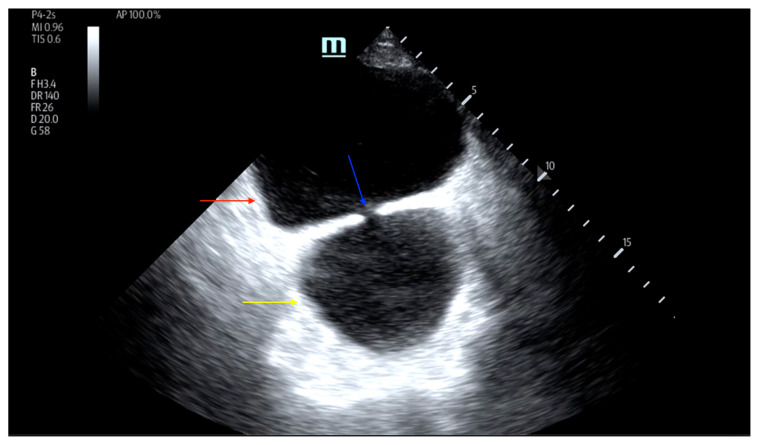
Video Link: https://youtu.be/3dkGxVDWED4

**Figure:**
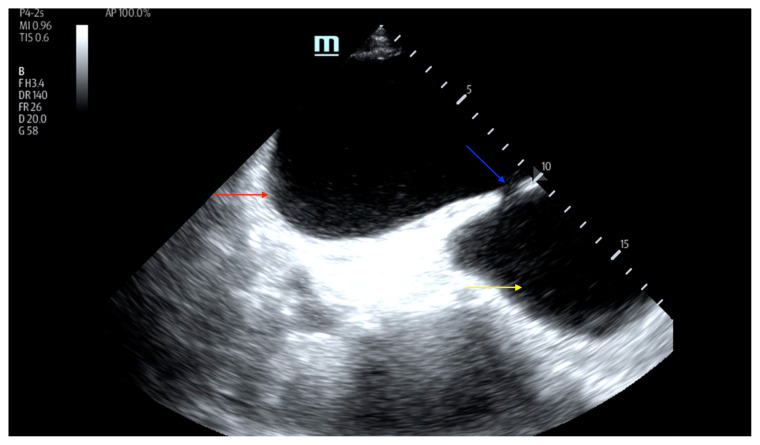
Video Link: https://youtu.be/SHWrMBn7n00

## Introduction

Bladder diverticula have previously been described as either congenital or acquired. Acquired causes include trauma, infection and outlet obstruction. Diverticula originate from points of weakness in the bladder wall that cause protrusion of the mucosa through the muscular wall.^1^ Diverticula findings are often incidental and secondary during workup of another presenting symptom. For example, benign prostatic hyperplasia (BPH) presents with bladder diverticula in 6% of cases with obstruction at the bladder vesical neck.^2^ Bladder diverticula have also been linked to more concerning sequelae or etiologies, such as carcinoma, perforation, and infection. Therefore, depending on clinical scenario, emergent urology consultation or ambulatory referral with urology to determine the cause of the diverticulum is imperative. Here we discuss the incidental finding of a rare anatomical presentation of a bladder diverticulum on POCUS during evaluation of urinary retention.

## Presenting concerns and clinical findings

A 76-year-old male presented to the emergency department with urinary retention, lower abdominal pain, and dysuria symptoms. He presented with complaint of not being able to void for the past fifteen hours since discharge from his initial ED visit for the same complaint. His urinary retention during the initial visit was relieved by in-&-out foley catheterization. He endorsed symptoms of difficulty urinating, dysuria, urgency, and frequency for the past three months. Patient’s past medical history was significant for abdominal hernia and atrial fibrillation. One month prior to his initial ED visit, he was started on tamsulosin (0.4 mg capsule daily) by his primary care physician for progressive difficulty urinating. On arrival to the ED, he was alert and oriented, but in mild distress. Complete review of systems was obtained and negative except as noted above. However, pertinent negatives include: fever, nausea, vomiting, diarrhea, hematuria, hematochezia. Vital signs were within normal limits. His medical, family, and social history were non-contributory.

## Significant findings

On examination, the patient was alert and oriented but in mild distress. Suprapubic fullness was noted upon abdominal palpation. Point of care ultrasound of the bladder showed two enlarged “bladders” with a central communication. Bedside total bladder volume was measured to be 1288 cm^3^ (the top “bladder” was measured to be 1011 cm^3^, while the bottom “diverticulum” was measured to be 277 cm^3^) by ultrasound. The POCUS stills of the patient’s bladder demonstrated the bladder (red arrow) and bladder diverticulum (yellow arrow) with a central communication (blue arrow) in the transverse and sagittal views.

## Patient course

The patient was determined to have been experiencing urinary retention secondary to benign prostatic hypertrophy. An indwelling foley catheter with a leg bag was inserted in the ED. The patient was discharged with the indwelling foley catheter with plans to follow up with urology in one week. During his appointment with urology, the patient endorsed one day of self-resolving gross hematuria following urinary catheterization. Upon physical exam, urology noted the catheter was draining clear urine. Patient was found to have an enlarged, symmetrical prostate that urology deemed benign. Urology discussed different medical and surgical treatment options to address BPH with the patient and agreed upon adding finasteride (5 mg tablet daily). Further urology workup included: obtaining a CT urography to evaluate patient’s hematuria, cystoscopy and trial voiding for evaluation of urinary retention, a urine culture to evaluate for urinary tract infection (UTI), and multichannel urodynamics procedure to determine bladder functionality. CT urography showed diffuse urothelial thickening possibly related to UTI, urinary bladder with large urinary bladder diverticulum, with moderate prostatomegaly, which may be related to chronic outlet obstruction, and incidental note of bilateral renal cysts, Bosniak type I and type II. Cystoscopy revealed no masses or stones, a large bladder diverticulum near the bladder neck, and demonstrated a hypoactive bladder with consequences of long-standing obstruction. The diverticulum close to the bladder neck was filled with infected urine, from which a culture was taken. After cystoscopy revealed no concerning etiologies for the patient’s urinary retention and bladder diverticulum, the in-&-out foley catheter was removed, and the patient was taught how to perform clean intermittent catheterization for management of his urinary retention. Urine culture showed enterococcus, for which patient was started on Levaquin. Urodynamic evaluation showed large bladder capacity with large residual urine volume, bladder voiding contraction of high amplitude, and bladder outlet obstruction grade 5/6. With all the results in mind, urology concluded that the patient’s bladder diverticulum was most likely due to longstanding obstruction as a result of BPH. The patient discussed the findings with his urologist and decided to undergo a transurethral resection of the prostate (TURP) to remedy his urinary retention and has been doing well since.

## Discussion

Though often an incidental finding, bladder diverticula should be appropriately scrutinized and screened for severe etiologies or consequences, such as cancer, nerve injury, or damage due to prior bladder surgery. A study examining the prevalence of bladder diverticula in cadavers found that 23.4% of cases had evidence of a urinary bladder diverticulum, with 14.3% of those cases showing malignant change.^3^ The most likely site of diverticulum is the area above and lateral to the ureteral orifice, which is characterized by absence of muscle fibers, which predisposes to pouch formation.^4^ These acquired bladder diverticula are susceptible to recurrent infections and stone formation due to stasis of urine. Ultimately, this can lead to squamous metaplasia of the bladder epithelium, subtly increasing the risk for squamous cell carcinoma of the bladder wall and urothelial carcinoma.^5^ Therefore, when bladder diverticula are discovered in a patient with symptoms concerning for cancer, such as weight loss, malaise, or unexplained gross hematuria, emergent follow up with urology is indicated. Iatrogenic causes of bladder diverticula are also a concern, leading to late complications if unrecognized. A case report of a patient presenting with irritative voiding symptoms and recurrent UTIs found a pseudotumor in an infected, obstructed bladder diverticulum caused by long-term retention and inflammation due to a misplaced suture after inguinal hernia repair.^6^ Unrecognized bladder injury after surgery can promote diverticula formation and can lead to pseudotumor development due to the chronic inflammatory response. When evaluating patients with urinary tract complaints and known bladder diverticula, it is imperative to be aware of a history of inguinal and abdominal surgery.

Alternatively, bladder diverticula can be a sequela of benign etiologies such as benign prostatic hyperplasia or bladder outlet obstruction. Such cases most commonly occur in elderly men and are asymptomatic, with an incidence range from 1 to 8%, and are discovered incidentally during investigation for other causes of BPH.^7^ Though publications about acquired bladder diverticula are scarce, interestingly, the widespread use of medical treatment for BPH may be a cause of elevated incidence of diverticula due to an increased period of obstruction, leading to an increase of bladder damage.^8^ In these cases, the diverticula can cause urine stasis and bladder wall damage, leading to complications including stone formation, UTIs, and tumors. Consequently, workup should include imaging, urinalysis, and cystoscopy to rule out these complications. Urinalysis can be done in the Emergency Department so that prompt recognition of a UTI and initiation of antibiotics can occur, while imaging and cystoscopy can be done outpatient.

Atypically, our patient presented with an inferior bladder diverticulum rather than lateral connected by a central canal to the normal bladder. Diverticulum formation was concluded to be a result of urinary retention and consequent bladder outlet obstruction secondary to BPH. Clinically, we had no concern of infection due to his physical exam and vital signs. In our case, the bladder diverticulum was not complicated by other presenting symptoms such as significant weight loss, hematuria, absent sensation, or urinary incontinence. Rather, it was found unintentionally during a POCUS evaluation for urinary retention. Regardless of the absence of other complications listed above, patient was scheduled for a follow up with urology in order to confirm the cause and screen for other concerning etiologies or complications, including carcinoma, nerve injury, or bladder damage. The extensive workup performed by urology, which included a CT urography, cystoscopy and trial voiding, urine culture, and urodynamic studies, did not show evidence of cancer or injury to the bladder. Therefore, urology concluded that the patient’s diverticulum was most likely a result of bladder outlet obstruction secondary to BPH, and the patient was able to undergo TURP procedure to address his urinary retention.

## Supplementary Information








