# Supplementary figures and images for: Bladder Diverticulum—A Case Report
Source: J Educ Teach Emerg Med. 2020 Oct 15;5(4):V15–8. doi: 10.21980/J8635C (PMC10332518; doi:10.21980/J8635C)

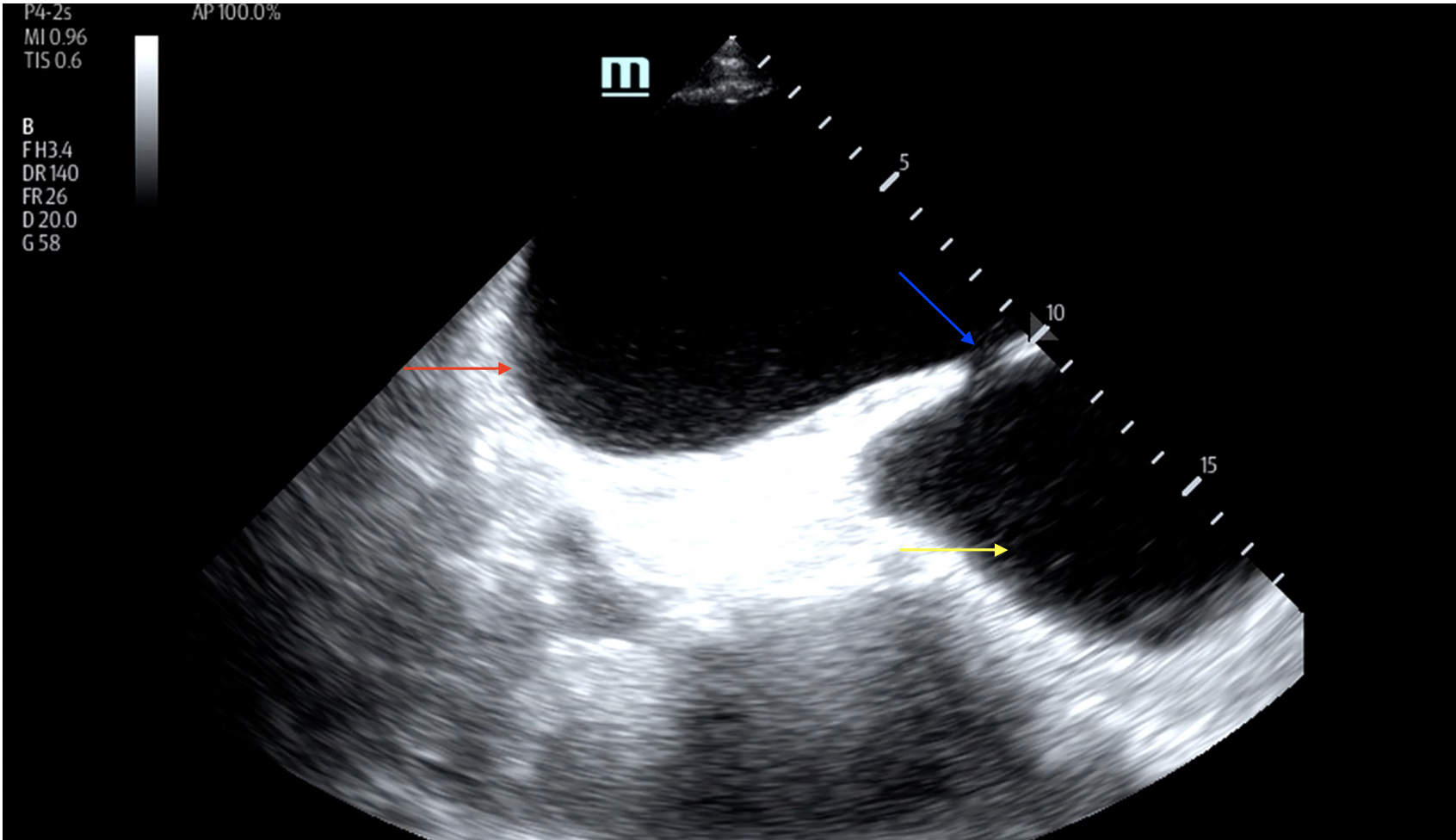

Supplement: Supplementary file 1 [file jetem-5-4-v15-supp1.jpg]

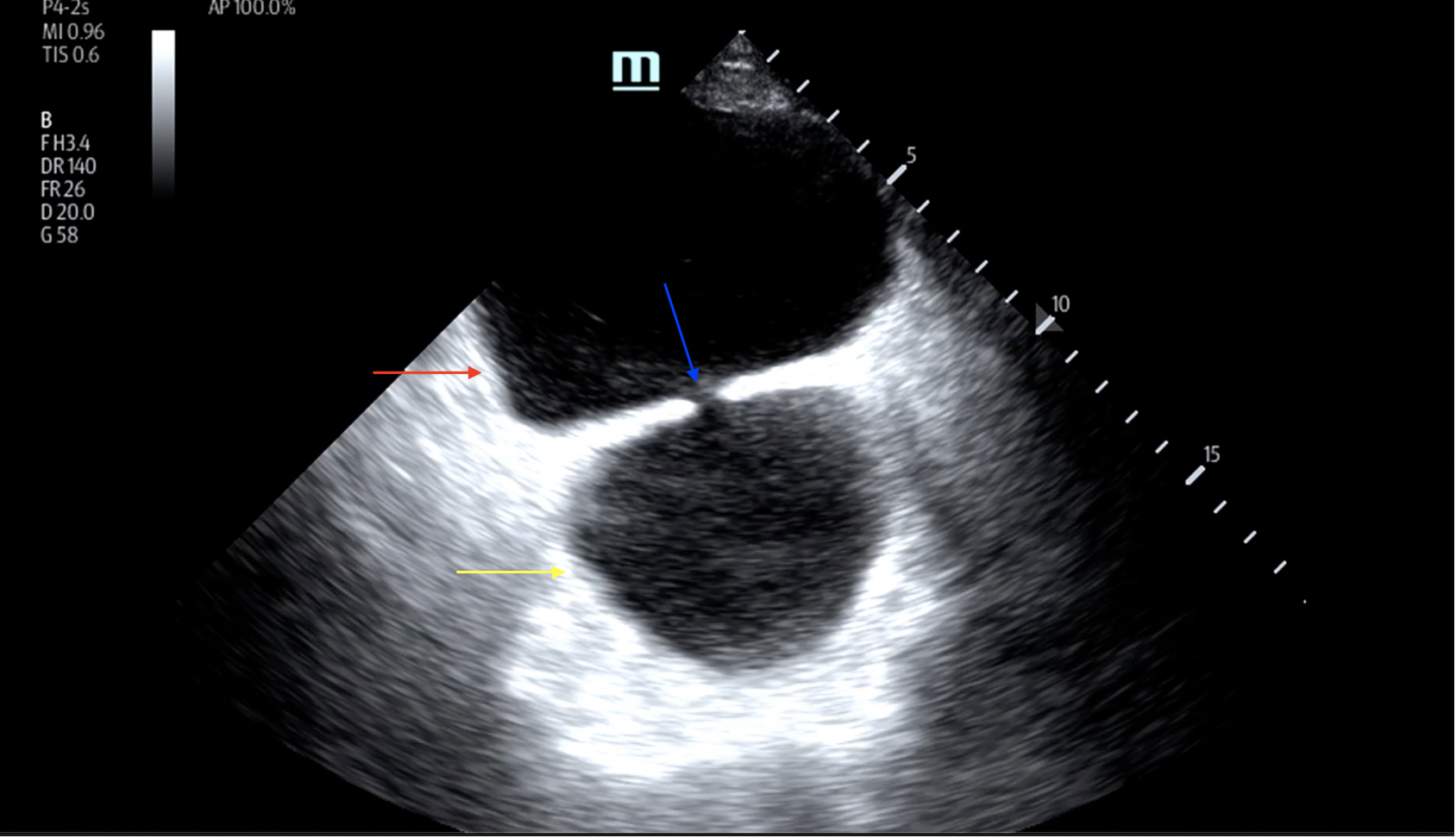

Supplement: Supplementary file 3 [file jetem-5-4-v15-supp3.jpg]
